# Diabetes risk among US adults with different socioeconomic status and behavioral lifestyles: evidence from the National Health and Nutrition Examination Survey

**DOI:** 10.3389/fpubh.2023.1197947

**Published:** 2023-08-22

**Authors:** Ce Liu, Li He, Yuanfei Li, Aimin Yang, Kai Zhang, Bin Luo

**Affiliations:** ^1^Institute of Occupational Health and Environmental Health, School of Public Health, Lanzhou University, Lanzhou, China; ^2^Department of Sociology, University at Albany, State University of New York, Albany, CA, United States; ^3^Hong Kong Institute of Diabetes and Obesity, The Chinese University of Hong Kong, Hong Kong, Hong Kong SAR, China; ^4^Department of Environmental Health Sciences, School of Public Health, University at Albany, State University of New York, Rensselaer, NY, United States

**Keywords:** socioeconomic status, access to healthcare, diabetes mellitus, behavioral factors, Mexican American

## Abstract

**Background:**

Diabetes disproportionately affects minorities and those with low socioeconomic status (SES) in the United States, and differences in behavioral lifestyles are largely responsible for the unequal distribution of diabetes among different groups.

**Methods:**

With data of 9,969 participants collected in the 2007–2008 and 2009–2010 cycles of the US National Health and Nutrition Examination Survey (NHANES), this study examined several mediators and their mediating effects in the connection between SES and the risk of diabetes. The SES is assessed by the income-to-poverty ratio (IPR), education level, and employment status. For the mediation analysis, we used health-related behaviors as mediators (smoking, alcohol use, consumption of green vegetables and fruits, physical activity and sedentary time, health insurance, and healthcare). In this study, the structural equation model was utilized to evaluate the mediating effects of behavioral lifestyle as a mediator in the relationship between SES and diabetes.

**Results:**

A total of 9,969 participants were included in this study. We found a negative nonlinear association between IPR and diabetes risk (*P*_overall_ < 0.001; *P*_non-linear_ = 0.46), which was independent of the majority of known or suspected risk factors and confounding variables (gender, age, race). Participants with lower SES had higher risk of diabetes compared with those with higher SES. In mediating analysis, we found alcohol intake (OR = 0.996), physical activity (OR = 0.993), health insurance (OR = 0.998), and healthcare (OR = 1.002) mediated the IPR-diabetes association. But in the relationship between education status and diabetes, the mediation effect of alcohol intake (OR = 0.995), physical activity (OR = 0.991), and health care (OR = 1.008) were obvious. Likewise, alcohol intake (OR = 0.996), fruit intake (OR = 0.998), and health care (OR = 0.975) were important mediators in the association between employment status and diabetes.

**Conclusion:**

This study provides critical insights on the link between SES and diabetes. Our results highlight that poor health-related behaviors and limited access to healthcare are important pathways for increased diabetes risk related to those with low SES, particularly among Mexican Americans and males. They should be top priorities for agencies and healthcare providers to develop behavior-related interventions to reduce inequalities in diabetes risk.

## 1. Introduction

Diabetes has increasingly become common because of fast economic growth and urbanization worldwide (1). According to the Global Burden of Disease Study, the global age-standardized prevalence of diabetes in 2019 was 5555.39 per 100,000 people, with an age-standardized mortality rate of 19.47 per 100,000 population (2).

Diabetes has emerged as a chronic non-communicable disease with a high disease burden and economic losses, making it a major public health problem affecting human health (3). It causes various adverse health problems, including blindness, visual impairment, kidney and lower extremity disease, acute metabolic complications, and increased cardiovascular and all-cause mortality (4).

In 2018, 34.2 million people in the U.S. were diagnosed with diabetes, and 88 million U.S. adults had a prediabetic status (5). However, the diabetes burden is unevenly distributed among the U.S. population. The incidence and prevalence of diabetes vary widely among people from different socioeconomic status (SES), and racial/ethnic groups (6). Environmental variables, behavioral factors, and distinct demographic features such as individuals of different genders and ages as well as people with different vocations have been studied in relation to diabetes (7, 8). SES is a crucial determinant in the uneven prevalence of diabetes, but its influence can be changed by the behavioral factors of an individual. There is some evidence suggesting disparities in the risk of getting diabetes and ultimate outcomes among people with various SES (9).

In line with Healthy People 2020, the US National Public Health Goals Blueprint advocates for strengthening diabetes-preventative behaviors, expanding access to effective lifestyle treatment approaches, and reducing diabetes-related socioeconomic health inequalities (10). Therefore, it is critical and urgent to find mediators of SES and diabetes to achieve this aim. Poor health-related behaviors and a lack of access to healthcare services have been possible mediators in the relationship between SES and diabetes (11). However, few studies have investigated the mediators of SES that influence the relationship between diabetes risk and outcomes. Therefore, this study aim to (1) identify mediators (including health-related behaviors, healthcare access, among others) linking SES and diabetes in the U.S. population, and (2) determine the extent to which the identified mediators contribute to the relationship between SES and diabetes in the U.S. population. Therefore, we explored the factors mediating the effect of SES on diabetes risk through health-related behaviors, with the aim of providing a scientific basis for developing diabetes prevention policies and control measures targeting vulnerable populations (e.g., those with low SES, those in prediabetes, and older populations).

## 2. Methods

### 2.1. Data sources

The data used for the analysis were obtained from the National Health and Nutrition Examination Survey (NHANES) (2007–2008 and 2009–2010) (12). The survey was conducted among participants aged 20 or over and confirmed not to be pregnant (*n* = 11,902). Excluding 1,150 individuals with missing income information and 783 individuals with missing information on fasting glucose, oral glucose tolerance test data, and glycated hemoglobin data, a total of 9,969 subjects were included in the analysis.

### 2.2. Study variables

#### 2.2.1. SES

Income, education, and occupation are commonly used to evaluate the SES of individuals and households (13). Therefore, we used three separate indicators, household income-to-poverty ratio (IPR), individual education level, and employment status to capture these three different dimensions of SES. IPR is calculated by dividing household income by the U.S. federal poverty line specific to that household size and year. PIR is adjusted for household size, composition and age of household members, and is updated annually for inflation (12). For analysis purposes (in order to maintain a sufficient number of participants in each category), the IPR was divided into three levels based on quartiles: lowest (≤1.36), middle (1.37–3.29), and highest (3.30–5.00) levels to maintain a sufficient number of participants in each category for analytical purposes (14). NHANES used five ordinal categories to describe educational levels: less than 9th grade, 9–11th grade, high school graduate, some college or associate in arts (AA) degree, and college graduate or higher. The employment status is a binary variable indicating whether an individual was employed or not.

#### 2.2.2. Health-related behaviors

Smoking, alcohol consumption, sedentary behavior, physical activity, consumption of green vegetables and fruits, health insurance, and healthcare usually vary across different SES populations and are all associated with health status (15). Smoking status was self-reported. For current smokers: participants reported having smoked at least 100 cigarettes in their lifetime and reported smoking every day or some days of the week at the time of the interview. Past smokers: participants reported having smoked at least 100 cigarettes in their lifetime, but not every day or some days of the week at the time of the interview. All others were defined as non-smokers (16). Because the rest of the tobacco use situations are very few and account for a negligible percentage of the total population of the study. Therefore, we only considered smoking status.

Alcohol consumption was assessed by asking the participants to quantify the number of alcoholic beverages they had consumed in the past 12 months, which includes three categories: (1) none or low (< 1 drink per month), (2) moderate (1–19 drinks per month), and (3) high (≥ 20 drinks per month) (17).

Participants were considered to have unhealthy consumption behavior of green vegetables and fruits if they reported no or rare intake of fruits or green vegetables at home (18). Participants reported information regarding their recreational physical activity, and those who reported moderate to vigorous recreational physical activity at least 3–5 times per week were considered to be physically active. Other participants were categorized as inactive.

Sedentary time was determined based on the number of hours per day spent sitting or lying at work, at home, or at school (excluding sleep time) and was classified as (1) low (≤ 1 h/day), (2) moderate (2–3 h/day), and (3) high (≥ 4 h/day) (19).

The following two aspects was used to evaluate healthcare access: (1) health insurance coverage and (2) self-reported healthcare usage (based on the number of times a participant received healthcare from a doctor or healthcare professional). Participants with a low SES were more likely to be without health insurance and face obstacles in pursuit of healthcare services compared with those with a high SES (20–22).

#### 2.2.3. Diabetes mellitus

The WHO diagnostic criteria for diabetes mellitus of 2021 was used to perform a diagnosis: a. Fasting plasma glucose ≥126 mg/dL, fasting was defined as no caloric intake for at least 8 h; b. Plasma glucose ≥200 mg/dL for 2 h during oral glucose tolerance test was conducted as described by the WHO guideline using a glucose load containing the equivalent of 75 g anhydrous glucose dissolved in water; c. HbA1c ≥ 6.5% (48 mmol/mol) (23).

### 2.3. Statistical analysis

Continuous variables were expressed as medians (interquartile distances), while categorical variables were expressed as percentages, and differences between SES groups were tested by Kruskal-Wallis tests and chi-square tests, respectively. A multivariate restricted cubic spline regression in R software (version 4.1.2; https://www.r-project.org/) was fitted through the “rms” package. The spline curves were used to test whether the regression had a linear or non-linear relationship and to determine the significance of this relationship. Analysis of multiple mediating effects models was performed through structural equation modeling (SEM) in the Stata software (version 16.0; https://www.stata.com/). The multiple mediating effects models were specified as follows:

(1) SEM


(1) Diabetes~Age+Gender+Race+c∗SES

(2) $$\mathrm{Health}-\mathrm{related behaviors}\sim \mathrm{Age}+\mathrm{Gender}+\mathrm{Race}\;\;+a_{i}*\mathrm{SES}$$

(3) $$\begin{array}{c} \mathrm{Diabetes}\sim \mathrm{Age}+\mathrm{Gender}+\mathrm{Race}+c^{\prime }*\mathrm{SES}+\overset{N}{\underset{i}{\sum }}b_{i}*\mathrm{Health}\\ -\mathrm{related behaviors} \end{array}$$

(2) Multiple mediating effects model.


(1) Total effect:c

(2) $$\;\mathrm{Sum}\;\mathrm{of mediating effect}:ab=\overset{N}{\underset{i}{\sum }}a_{i}*b_{i}$$

(3) $$\mathrm{Direct effect}:c^{\prime }=c-ab$$

(4) $$\mathrm{The proportion of mediating effect}:\frac{a_{i}*b_{i}}{c}$$

(5) $$\mathrm{OR value of each mediator}:\mathrm{OR}=\exp\left(a_{i}*b_{i}\right)$$

Health-related behaviors include the following eight items: alcohol intake, smoking, fruit consumption, green vegetable consumption, health insurance, health care, physical activity, and sedentary time. A Bootstrap method with 5,000 self-help extractions was adopted to calculate the confidence intervals. The mediating effect of each mediating pathway from the SES to diabetes was assessed using the coefficient product method. The OR value reflects the relationship between SES and the risk of diabetes through a mediator path (Supplementary Figure S2). If the OR of a mediating factor is below 1, a negative correlation could be inferred between SES and the risk of diabetes through the mediator path. On the contrary, if the OR of a mediating factor is above 1, there would be a positive correlation between SES and the risk of diabetes through this path. The statistically significant difference was set at a two-sided *p* value of 0.05.

In this study, we also conducted mediating analysis for subgroups based on participants’ age, gender, and race/ethnicity. For a certain subgroup, however, we only estimated the mediating role of a health-related behavior when it shows significant interaction with participants’ SES.

## 3. Results

### 3.1. Baseline characteristics

The baseline characteristics of the study participants divided by the IPR are presented in Supplementary Table S1. It can be seen that more younger people and women had low IPR. Non-Hispanic whites accounted for the highest proportion of participants with high IPR whereas most participants with low IPR were non-Hispanic blacks and Mexican Americans. The proportion of participants with health-related risk behaviors was higher among those with low IPR. The same was true for the absence of health insurance and healthcare in the past year.

Supplementary Table S2 displays the baseline characteristics of the participants based on education level. The proportion of the older adult with low education was considerably large. Non-Hispanic whites had the highest share among the high-level education group whereas Mexican Americans constituted a large part of the low-level education group. Supplementary Table S3 shows the baseline characteristics by employment status of the study population. More older adults and women were unemployed.

### 3.2. The relationship between SES and the prevalence of diabetes

**Figure 1:**
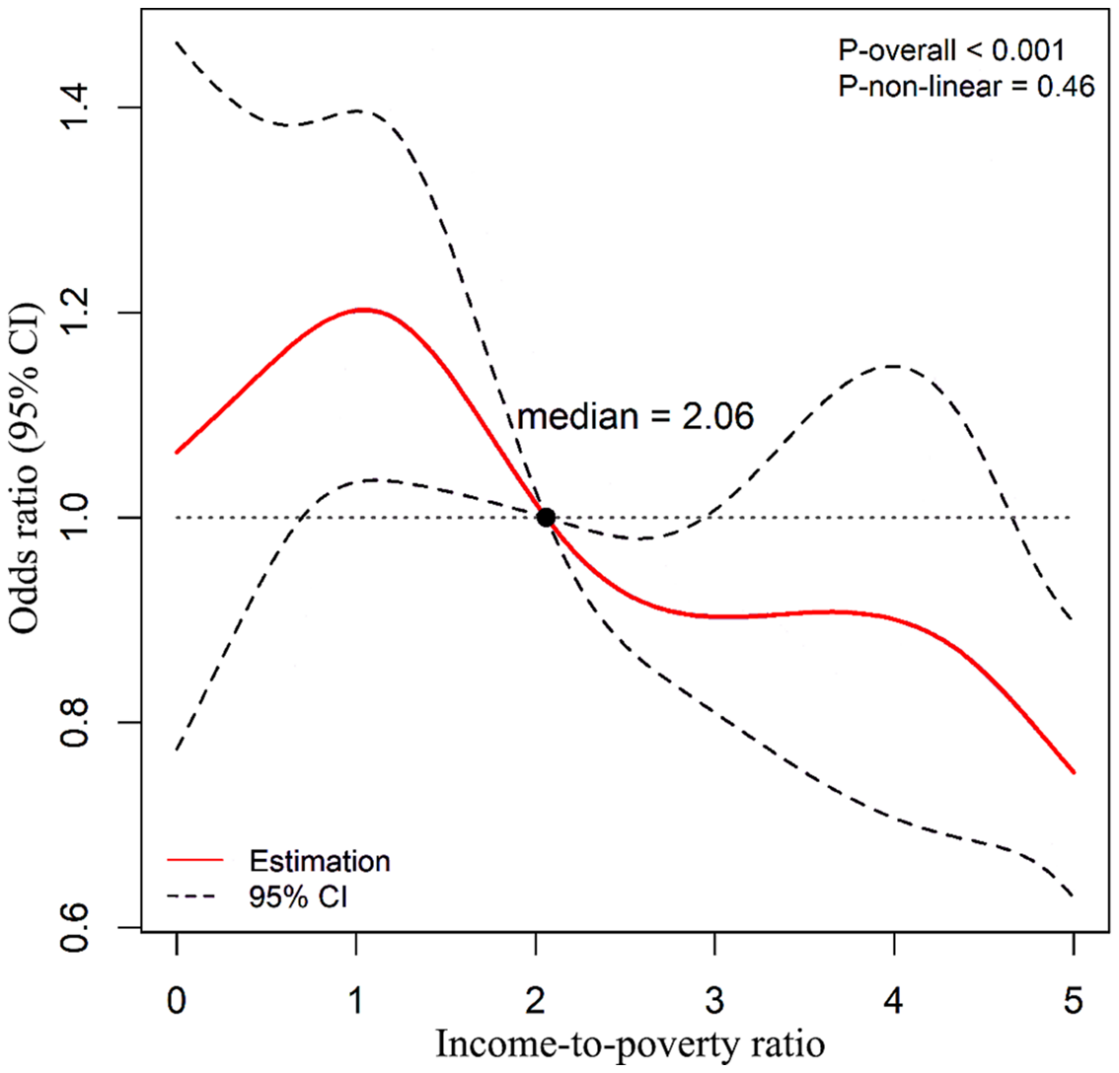
Restricted cubic spline regression model fitting the relationship between income-to-poverty ratio and diabetes risk. Confounding variables such as age, gender, and race were adjusted in the model. The reference value of ORs was the median of income-to- poverty ratio (2.06); the solid red line represents the OR, and the black dashed line represents the 95% CI of the OR; *P*_overall_ < 0.001; *P*_non-linear_ = 0.46.

Figure 1 shows the relationship between IPR and diabetes fitted by the restrictive cubic spline regression model. Before the IPR reached the median value (about 2.06), the risk of diabetes showed an inverted “V” trend as the IPR increased, and a relatively gentle downward trend was observed when the IPR exceeded 2.06 (Figure 1; *P*_overall_ > 0.001).

### 3.3. SES, diabetes, and mediating factors

Further analysis revealed a negative association between the odds of diabetes and IPR (OR = 0.960, 95% CI: 0.938, 0.983). Health-related behaviors, like alcohol intake (OR = 0.996, 95% CI: 0.991, 0.999), physical activity (OR = 0.993, 95% CI: 0.989, 0.998), health insurance (OR = 0.998, 95% CI: 0.995, 0.999), and healthcare (OR = 1.002, 95% CI: 1.001, 1.003) pathways were found to be important factors mediating the association. The mediating effects of smoking, sedentary time, and consumption of green vegetables and fruits were not statistically significant.

**Figure 2:**
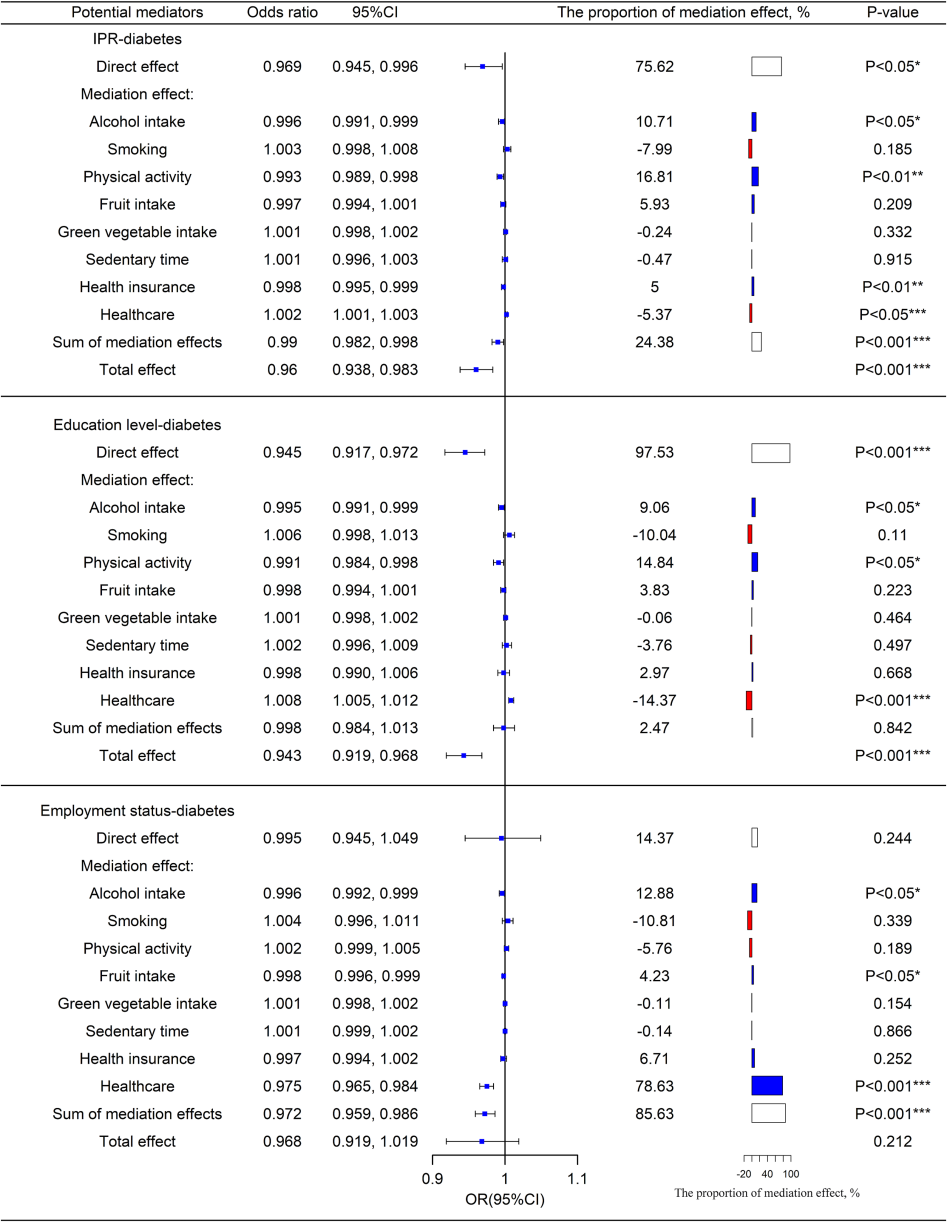
Mediating and direct effects of SES on diabetes. The proportion of the direct effect and the proportion of the sum of the mediation effect is shown in white, the proportion of the mediation effect with OR < 1 is shown in blue, and the proportion of the mediation effect with OR > 1 is shown in red. OR = exp. (a_i_*b_i_). OR < 1 means that improving SES can reduce the risk of diabetes through this mediating factor pathway; conversely, OR > 1 means that improving SES can increase the risk of developing diabetes through this mediating factor pathway. ^*^p < 0 0.05, ^**^p < 0.01, ^***^p < 0.001.

Figure 2 indicates that the risk of diabetes decreased as education levels increased (OR = 0.945, 95% CI: 0.917, 0.972). Alcohol intake (OR = 0.995, 95% CI: 0.991, 0.999), physical activity (OR = 0.991, 95% CI: 0.984, 0.998), and healthcare (OR = 1.008, 95% CI: 1.005, 1.012) exhibited significant mediating effects on the association between education and diabetes. Smoking, sedentary time, health insurance, and consumption of green vegetables and fruits were not significant factors in this case.

It was also found that health-related behaviors such as alcohol intake (OR = 0.996, 95% CI: 0.992, 0.999), fruit intake (OR = 0.998, 95% CI: 0.996, 0.999), and healthcare (OR = 0.975, 95% CI: 0.965, 0.984) were important mediators of the association between employment status and diabetes. In contrast, smoking, sedentary time, health insurance, and vegetable intake had no significant mediating effects on the relationship.

### 3.4. Interactions of SES with gender, race, and Age

The results also indicated that the correlation between SES and diabetes varied by gender, race, and age, suggesting some degree of interaction among them (Supplementary Figures S3–S5). Therefore, a mediation analysis stratified by gender, race, and age was performed.

### 3.5. Race subgroup analysis of SES-diabetes mediating effect

**Figure 3:**
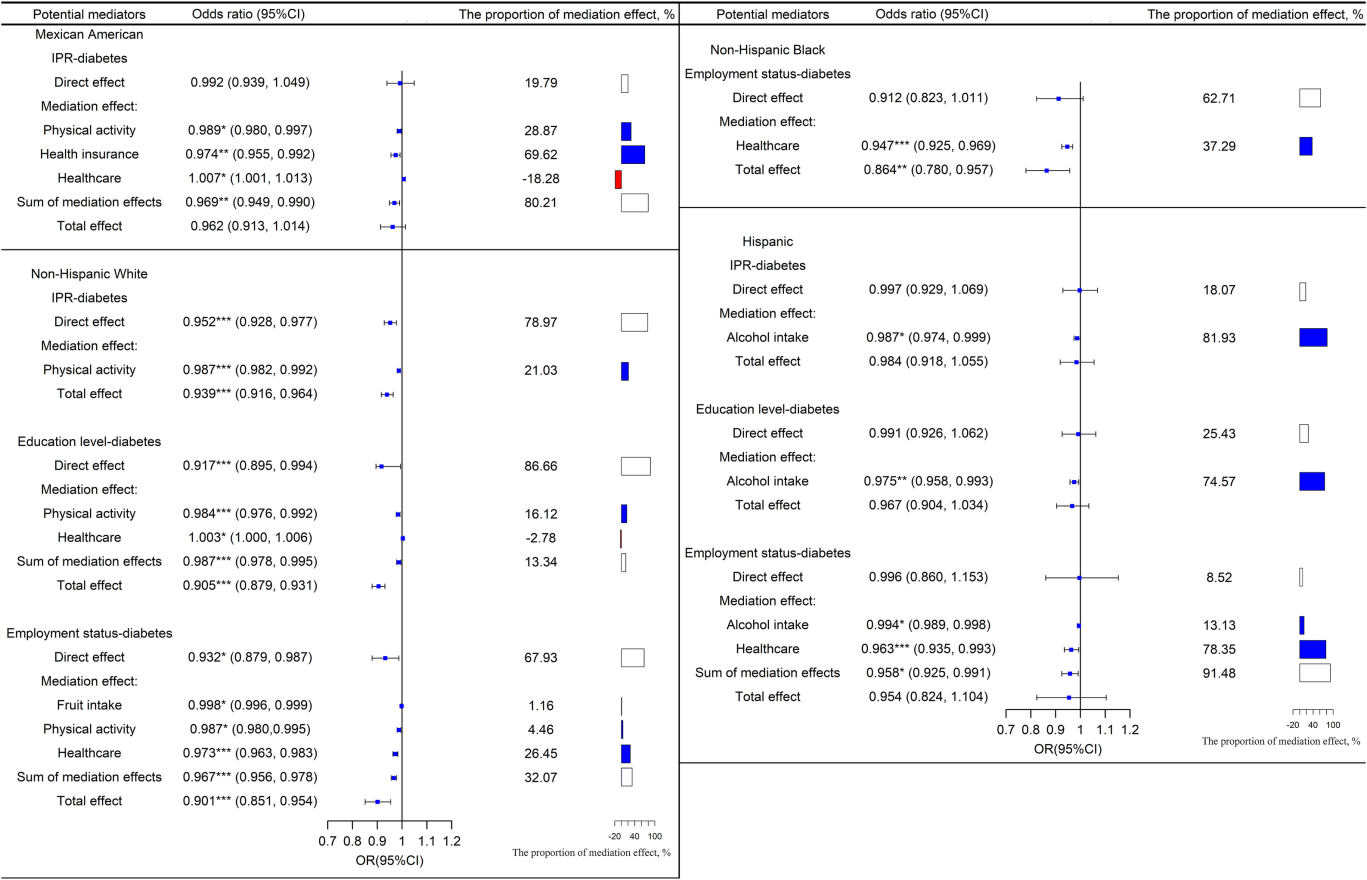
Mediation and direct effects of SES on diabetes in Mexican Americans, non-Hispanic white, non-Hispanic black, and Hispanics. The proportion of the direct effect and the proportion of the sum of the mediation effect is shown in white, the proportion of the mediation effect with OR < 1 is shown in blue, and the proportion of the mediation effect with OR > 1 is shown in red. OR = exp. (a_i_*b_i_). OR < 1 means that improving SES can reduce the risk of diabetes through this mediating factor pathway; conversely, OR > 1 means that improving SES can increase the risk of developing diabetes through this mediating factor pathway. ^*^p < 0 0.05, ^**^p < 0.01, ^***^p < 0.001.

Figure 3 displays the mediation effects and percentages of mediation for numerous potential mediators of the IPR-diabetes relationship among different races. In the Mexican American population, physical activity (OR = 0.989, 95% CI: 0.980, 0.997), health insurance (OR = 0.974, 95% CI: 0.955, 0.992), and healthcare (OR = 1.007, 95% CI: 1.001, 1.013) mediated the effect of IPR on diabetes. Physical activity (OR = 0.987, 95% CI: 0.982, 0.992) and alcohol intake (OR = 0.987, 95% CI: 0.974, 0.999) mediated the association between IPR and diabetes in non-Hispanic whites and Hispanic populations, respectively. However, none of the selected variables significantly mediated the IPR-diabetes relationship among non-Hispanic blacks and other races, including the multiracial population.

Figure 3 depicts the mediation effects and percentages for various potential mediators of the education-diabetes relationship across races. None of the selected factors significantly mediated the education-diabetes relationship among Mexican Americans, non-Hispanic blacks, and other races, including multiracial groups. However, among non-Hispanic whites, physical activity (OR = 0.984, 95% CI: 0.976, 0.992), and healthcare (OR = 1.003, 95% CI: 1.000, 1.006) mediated the association between education and diabetes. In the Hispanic population, alcohol intake (OR = 0.975, 95% CI: 0.958, 0.993) mediated the association between education and diabetes.

The mediation effects and percentages of mediation for potential mediators of the employment status-diabetes connection among different races are shown in Figure 3. None of the selected mediator variables showed a significant mediating effect on the education-diabetes association in Mexican Americans and other races, including multiracial populations. However, among non-Hispanic whites, fruit intake (OR = 0.998, 95% CI: 0.996, 0.999), physical activity (OR = 0.987, 95% CI: 0.980, 0.995), and healthcare (OR = 0.973, 95% CI: 0.963, 0.983) were found to be important mediators. Alcohol intake (OR = 0.994, 95% CI: 0.989, 0.998) and healthcare (OR = 0.963, 95% CI: 0.935, 0.993) also mediated the association between employment and diabetes in the Hispanic population.

### 3.6. Gender subgroup analysis of SES-diabetes mediating effect

**Figure 4:**
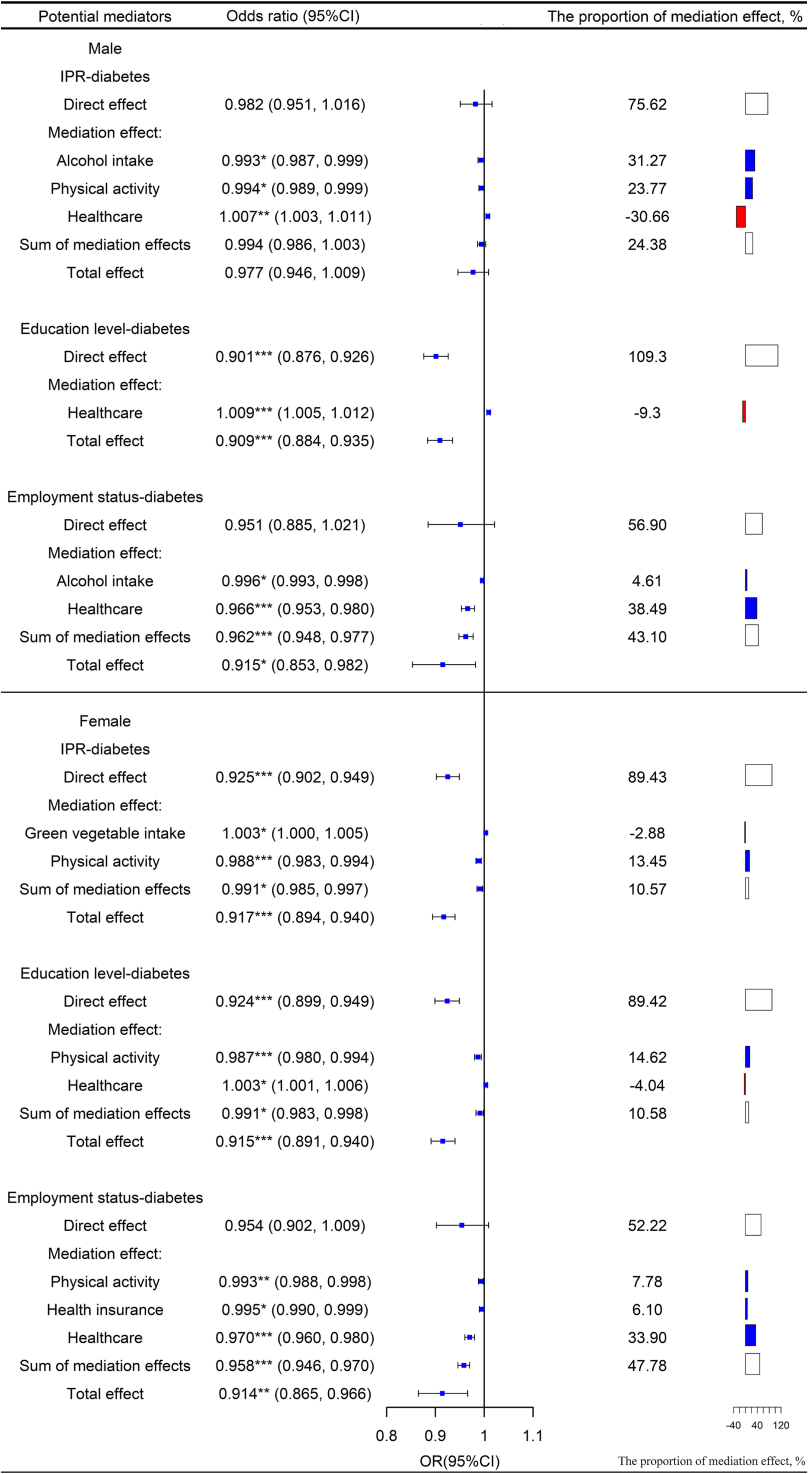
Mediation and direct effects of SES on diabetes in males and females. The proportion of the direct effect and the proportion of the sum of the mediation effect is shown in white, the proportion of the mediation effect with OR < 1 is shown in blue, and the proportion of the mediation effect with OR > 1 is shown in red. OR = exp. (a_i_*b_i_). OR < 1 means that improving SES can reduce the risk of diabetes through this mediating factor pathway; conversely, OR > 1 means that improving SES can increase the risk of developing diabetes through this mediating factor pathway. ^*^p < 0.05, ^**^p < 0.01, ^***^p < 0.001.

In terms of gender, the mediating effects and percentages of mediation for various potential mediators of the IPR-diabetes association are shown in Figure 4. In the female population, the overall contribution of identified significant health-related behaviors to the association between IPR and diabetes was 10.57%. Physical activity (OR = 0.988, 95% CI: 0.983, 0.994) was the main mediating factor. The direct effect of IPR on diabetes was greater in women than in men (women: OR = 0.925; men: OR = 0.982). In the male population, the overall contribution of identified health-related behaviors on the association between IPR and diabetes was 24.38%. Notably, alcohol intake (OR = 0.993, 95% CI: 0.987, 0.999) was the main mediating factor.

Similarly, the mediating effects and percentages of mediation for various potential mediators of the education-diabetes association for different genders are shown in Figure 4. Among females, the overall contribution of the identified significant health-related behaviors to the association between education and diabetes was 10.58%. Of these mediators, physical activity (OR = 0.987, 95% CI: 0.980, 0.994) contributed 13.45% to the mediation. The direct effect of education on diabetes was significantly lower in women than in men (women: OR = 0.924; men: OR = 0.901).

Mediating effects and percentages of mediation for various potential mediators of the employment-diabetes association are shown in Figure 4 by gender. In the female population, the overall contribution of identified significant health-related behaviors to the association between employment status and diabetes was 47.78%. Among them, both physical activity (OR = 0.993, 95% CI: 0.988, 0.998) and health insurance (OR = 0.995, 95% CI: 0.990, 0.999) played important mediating roles.

In the male population, the overall contribution of identified significant health-related behaviors on the association between employment status and diabetes was 43.10%. Among the behavioral factors, alcohol intake (OR = 0.996, 95% CI: 0.993, 0.998) played some mediating role. In both male and female populations, the direct effect of employment status on the risk of diabetes was not significant.

### 3.7. Age subgroup analysis of SES-diabetes mediating effect

**Figure 5:**
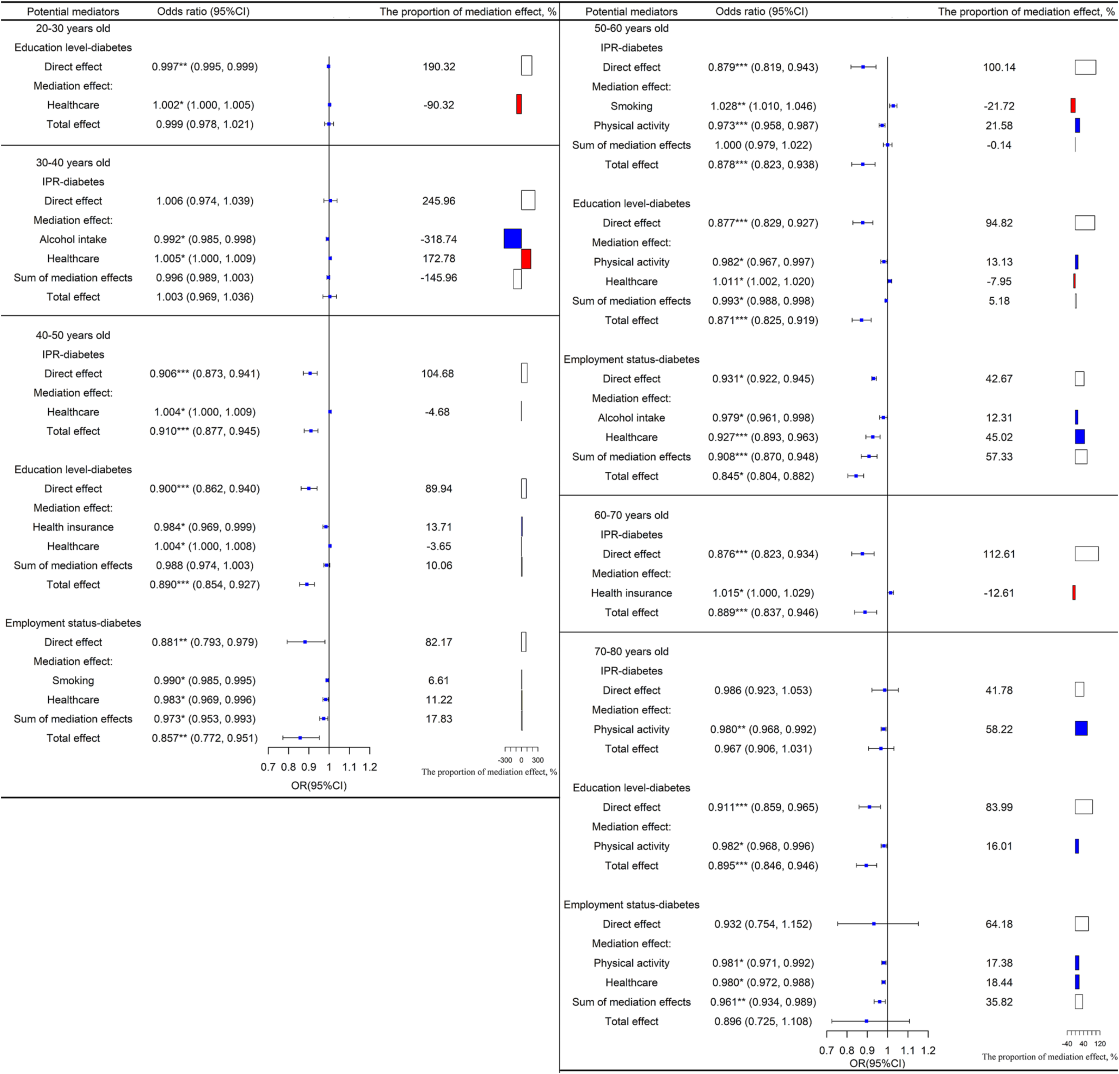
Mediation and direct effects of SES on diabetes in people aged 20–30, 30–40, 40–50, 50–60, 60–70, 70–80. The proportion of the direct effect and the proportion of the sum of the mediation effect is shown in white, the proportion of the mediation effect with OR < 1 is shown in blue, and the proportion of the mediation effect with OR > 1 is shown in red. OR = exp. (a_i_*b_i_). OR < 1 means that improving SES can reduce the risk of diabetes through this mediating factor pathway; conversely, OR > 1 means that improving SES can increase the risk of developing diabetes through this mediating factor pathway. ^*^p < 0.05, ^**^p < 0.01, ^***^p < 0.001.

Mediation effects and percentages of mediation for various potential mediators of the IPR-diabetes association are shown in Figure 5 by age. In the group aged 30–40 years, reducing alcohol intake was the main pathway for increasing IPR to reduce the risk of diabetes (OR = 0.992, 95% CI: 0.985, 0.998). Among people aged 40–50 years, the risk of diabetes was reduced through the direct effect of improved IPR (OR = 0.906, 95% CI: 0.873, 0.941). In people aged 50–60 years, physical activity (OR = 0.973, 95% CI: 0.958, 0.987) was the main pathway for increasing IPR to reduce the risk of diabetes, though the direct effect of IPR also accounted for a large proportion of the total effect (OR = 0.879, 95% CI: 0.819, 0.943).

In terms of age, the mediation effects and the percentages of several mediators of the education-diabetes relationship are shown in Figure 5. Education had a strong direct effect on diabetes risk in all age groups (20–30 years old: OR = 0.997, 95% CI: 0.995, 0.999; 40–50 years old: OR = 0.900, 95% CI: 0.862, 0.940; 50–60 years old: OR = 0.877, 95% CI: 0.829, 0.927; 70–80 years old: OR = 0.911, 95% CI: 0.859, 0.965). Physical activity was the most important factor mediating the association between education and diabetes in people aged 50–60 years and 70–80 years (50–60 years old: OR = 0.982, 95% CI: 0.967, 0.997; 70–80 years old: OR = 0.982, 95% CI: 0.968, 0.996).

Figure 5 depicts the mediation effects and the percentages of the selected mediators on the relationship between employment and diabetes by age. Smoking was an important factor mediating the relationship between employment status and diabetes in people aged 40–50 years (OR = 0.990, 95% CI: 0.985, 0.995). Similarly, alcohol intake mediated the relationship between employment status and diabetes among people aged 50–60 years (OR = 0.979, 95% CI: 0.961, 0.998). The results showed that physical activity mediated the association between employment status and diabetes in people aged 70–80 years (OR = 0.981, 95% CI: 0.971, 0.992).

## 4. Discussion

This study explored the factors that may influence the relationship between SES and diabetes by using the nationally representative NHANES dataset in the United States. Our results indicate that behaviors such as alcohol intake, smoking, physical activity, health insurance, and healthcare are important in mediating the SES-diabetes relationship. This is especially relevant for males and Mexican Americans compared with other groups. Thus we contribute new insights into the mechanisms affecting diabetes risks.

A higher SES indicates better levels of IPR, education, and occupation. This study found a negative association between SES and diabetes risk. A lower level of IPR is an important risk factor for diabetes. This may be related to the challenges of maintaining healthy dietary habits and good dietary quality among people with lower income levels (24, 25). In addition to diet, the lack of sufficient health care services to address diabetes issues is also an important factor. In a diabetes survey in 2010, about 9.1% of the interviewed American adults did not have health insurance, which was linked to a high risk of diabetes (26). A previous study also had similar findings, that diabetic patients with lower SES had a mortality risk that was often twice as high as those with higher SES (27). These factors may help explain why a lower level of IPR is an important risk factor for diabetes.

Behavioral lifestyles differ substantially across SES levels. Furthermore, with the same level of health intention, people with higher SES levels may face fewer challenges in achieving healthier behavioral lifestyles (28). Smoking, alcohol intake, consumption of green vegetables and fruits, physical activity, sedentary time, and healthcare are the main factors in behavioral lifestyle that influence the development of diabetes (29), which together are related to the higher risk of diabetes onset (30). Therefore, these behaviors may mediate the effect of SES on diabetes.

Results showed that alcohol consumption, physical activity, health insurance, and healthcare contributed 10.71, 16.81, 5%, and − 5.37% to the total effect of IPR on diabetes, respectively. Through the SES-Alcohol-Diabetes pathway, a higher SES was associated with a lower level of alcohol consumption, and lower risk of diabetes (31). Among these four factors, physical activity mediated the most effect of SES on diabetes risk, and a higher SES is positively related to increased physical activity that could lower diabetes risk. The increase in physical activity would increase the energy expenditure and promote insulin sensitivity, which in turn reduces the diabetes risk (32).

Additionally, we found that health insurance also mediated the relationship between SES and diabetes. This is because people with higher SES levels may have more ease in affording health insurance costs, and this may be related to a lower diabetes risk (33). Indeed, insured adults are significantly more likely to receive healthcare services for early detection of diabetes, which may reduce the diabetes risk (34).

There is a positive association between healthcare and SES, suggesting that people with higher SES have more access to healthcare and show a lower risk of diabetes (35). Therefore, healthcare is expected to be a potential mediator for the SES-diabetes relationship. However, we found a masking effect of healthcare in the effect of SES on diabetes risk. The positive association of healthcare and diabetes risk suggested that a high frequency of physical exams or healthcare may be associated with an increased risk of diabetes. Healthcare itself does not increase the risk of diabetes, and people with a high risk of diabetes have more healthcare needs (36). This leads to a positive correlation between healthcare and the risk of diabetes, which in turn indirectly suppresses the effect of SES on diabetes through the SES-healthcare-diabetes pathway (14). Studies reported that the lower quality of healthcare increased the risk of diabetes complications and mortality (37). In this study, the healthcare was only measured by the frequency of physical exams and not the healthcare quality because of the data limitation, and this may lead to this masking effect. Therefore, further studies should develop an index for assessing healthcare quality and explore the mediating effect of healthcare on the SES-diabetes relationship.

The mediating effect on the association between SES and diabetes was more prevalent among Mexican Americans than other racial groups, particularly for factors related to healthcare access. It is likely that this is caused by the strong association between SES and factors affecting access to healthcare among Mexican Americans and that the majority of Mexican Americans have limited access to healthcare (38).

Language and economic barriers and low education limit Mexican Americans’ health care services. In addition, racial consistency between doctors and patients is considered to improve patient satisfaction and service utilization (39). However, this consistency is usually a challenge because the number of Mexican patients often exceeds the number of available Mexican doctors (40). The low economic level also limits Mexican Americans’ health insurance coverage rate (41). These reasons have led to low health care services for Mexican Americans. Therefore, increasing medicare coverage in the Mexican American population can effectively reduce the risk of diabetes in the Mexican American population with low SES.

Additionally, we also found strong mediating effects of certain behaviors in the SES-diabetes relation among males. Specifically, alcohol consumption was found to be the most important mediating factor in the male population. Generally, alcohol consumption is more prevalent and more negatively affected by SES in males than in females (42). This is consistent with the findings of a previous meta-analysis on the association between SES and the risk of death from alcohol consumption (43). Therefore, in the male population, controlling alcohol intake should be an effective way to reduce the risk of diabetes in low SES groups.

However, it should be noted that physical activity cannot be ignored. The mediating effect of physical activity on the association between SES and diabetes in women was stronger than that of men, which may be explained by the stronger positive correlation between physical activity and SES found among women (44). These findings suggest that promoting physical activity should be considered an effective intervention to reduce the risk of diabetes, particularly in women with low SES (45).

Besides, alcohol consumption acted as an important mediating factor in the group aged 30–40 years compared with other age groups. Alcohol consumption was more prevalent in the younger population, which is also more affected by SES (46). This may lead to the fact that alcohol consumption has become an important mediator of SES affecting the risk of diabetes in people aged 30–40 (47).

Physical activity is the primary mediating factor in the 50–60 and 70–80 age groups. This could be because older people are more physically inactive than younger people, particularly those with lower SES (48). This results in a stronger relationship between SES and physical activity in older age groups (49). Among individuals aged 50–60 years old, smoking is a key masking factor. Middle-aged people usually face greater social pressures, such as work pressure, family burden, personal health, housing pressure, etc. (50, 51), and greater resistance to quitting smoking (52), which may increase the likelihood of smoking (53). Therefore, the smoking rate is higher among middle-aged people. Due to the increase in cigarette prices in recent years, smoking frequency and intensity decreased among middle-aged people with low SES. However, this phenomenon was not observed in middle-aged people with higher SES (54), thus their smoking intensity is more likely to increase with the increase in SES. These two reasons may likely explain why smoking is an important masking factor affecting the relationship between SES and diabetes risk in the 50–60 years old population. This masking effect may undercut the benefits of increased SES in reducing the risk of diabetes. Among people aged 50–60 years, smoking control is thus particularly important for reducing the risk of diabetes.

### 4.1. Strengths and limitations

This research had three major strengths. First, to the best of our knowledge, it is the first study to systematically examine the mediating role of the SES-diabetes association through health-related behaviors and healthcare as well as to estimate the effect size of these factors in the association. Data utilized in the current study were from a national sample of the U.S. adult population; hence, the results were adequately representative.

Second, the present study used SEM to analyze the mediation effect on the SES-diabetes relationship, which reduces the potential bias present in the traditional mediation analysis proposed by Baron and Kenny (55, 56). Finally, unlike previous studies (11), this study increased the precision of the degree of mediation by providing the proportion of the mediation effect.

This study also had some limitations that warrant further investigation. First, the cross-sectional nature of the NHANES data does not allow longitudinal assessment of mediators that may be more appropriate to explain the SES-diabetes association and especially for mediators that vary over time. Furthermore, we did not analyze psychological factors, such as anxiety and depression. This may be problematic because some studies suggest that poor mental health is also an important mediator of the association between SES and disease (57). In addition, poor mental health can have a serious impact on health-related behaviors (58). Finally, when considering physical activity as a factor, we were unable to specifically consider the type and mode of physical activity, such as a combination of aerobic and anaerobic activities or a single mode, due to data limitations. This resulted in a crude categorization of physical activity to explore its mediating effect in this study. Nevertheless, this study still has important public health and policy implications. By understanding the mediators of the relationship between SES and diabetes, it is possible to design interventions targeting at modifiable health-related behaviors and healthcare to reduce the risk of diabetes due to inequality in SES, especially among Mexican Americans.

## 5. Conclusion

This study provides further evidence for the association between SES inequality and diabetes risk and shows that poor health-related behaviors and limited access to healthcare are the pathways by which low SES may contribute to diabetes. In the United States, especially among Mexican Americans and males, the identified mediators contribute greatly to the association between low SES and diabetes, and they may be the appropriate population to implement interventions aimed at reducing the disparities in the risk of diabetes. Unlike disparities in SES, which were found to be the root cause and structural determinant of health inequities and are thus more difficult to change, behavioral lifestyle, which is a key mediator of SES in influencing disease development, is a more easily amenable factor of health inequalities. Therefore, improving behavioral lifestyles in low SES populations is an effective intervention to prevent diabetes and achieve health equity.

## Data availability statement

The original contributions presented in the study are included in the article/Supplementary material, further inquiries can be directed to the corresponding authors.

## Author contributions

CL: conceptualization, methodology, software, writing − original draft, and writing − review and editing. LH: formal analysis, visualization, and writing − review and editing. YL: methodology and writing − review and editing. AY: software, supervision, and writing − review and editing. KZ: conceptualization, writing − review and editing, and supervision. BL: conceptualization, methodology, funding acquisition, writing − review and editing, and supervision. All authors contributed to the article and approved the submitted version.

## Funding

This work was supported by the Key Research and Development Program of Gansu Province (grant number 20YF2FA028) and the Fundamental Research Funds for the Central Universities, Lanzhou University, China (grant number lzujbky-2021-ey07). The funders of this study had no role in the study design; the collection, analysis, and interpretation of data; the writing of the report; or the decision to submit the report for publication.

## Conflict of interest

The authors declare that the research was conducted in the absence of any commercial or financial relationships that could be construed as a potential conflict of interest.

## Publisher’s note

All claims expressed in this article are solely those of the authors and do not necessarily represent those of their affiliated organizations, or those of the publisher, the editors and the reviewers. Any product that may be evaluated in this article, or claim that may be made by its manufacturer, is not guaranteed or endorsed by the publisher.
