# Supplementary material for: Diabetes risk among US adults with different socioeconomic status and behavioral lifestyles: evidence from the National Health and Nutrition Examination Survey
Source: Front Public Health. 2023 Aug 22;11:1197947. doi: 10.3389/fpubh.2023.1197947 (PMC10477368; doi:10.3389/fpubh.2023.1197947)
Supplement: Supplementary file 1 [file Data_Sheet_1.docx]

Supplementary Material

Diabetes Risk Among US Adults with different Socioeconomic Status and Behavioral Lifestyles: Evidences from the National Health and Nutrition Examination Survey

**Ce Liu^1^, Li He^1^, Yuanfei Li^2^, Aimin Yang^3^, Kai Zhang^4*†^, Bin Luo^1*†^**

*** Correspondence:**

Bin Luo, PhD, Institute of Occupational Health and Environmental Health, School of Public Health, Lanzhou University, Donggang Xi Road 199, Lanzhou 730020, China ([luob@lzu.edu.cn](mailto:luob@lzu.edu.cn)).

Kai Zhang, PhD, Department of Environmental Health Sciences, School of Public Health, University at Albany, State University of New York, One University Place, Rensselaer, NY 12144, USA ([kzhang9@albany.edu](mailto:kzhang9@albany.edu)).

# Supplementary Figures and Tables

## Supplementary Figures

**
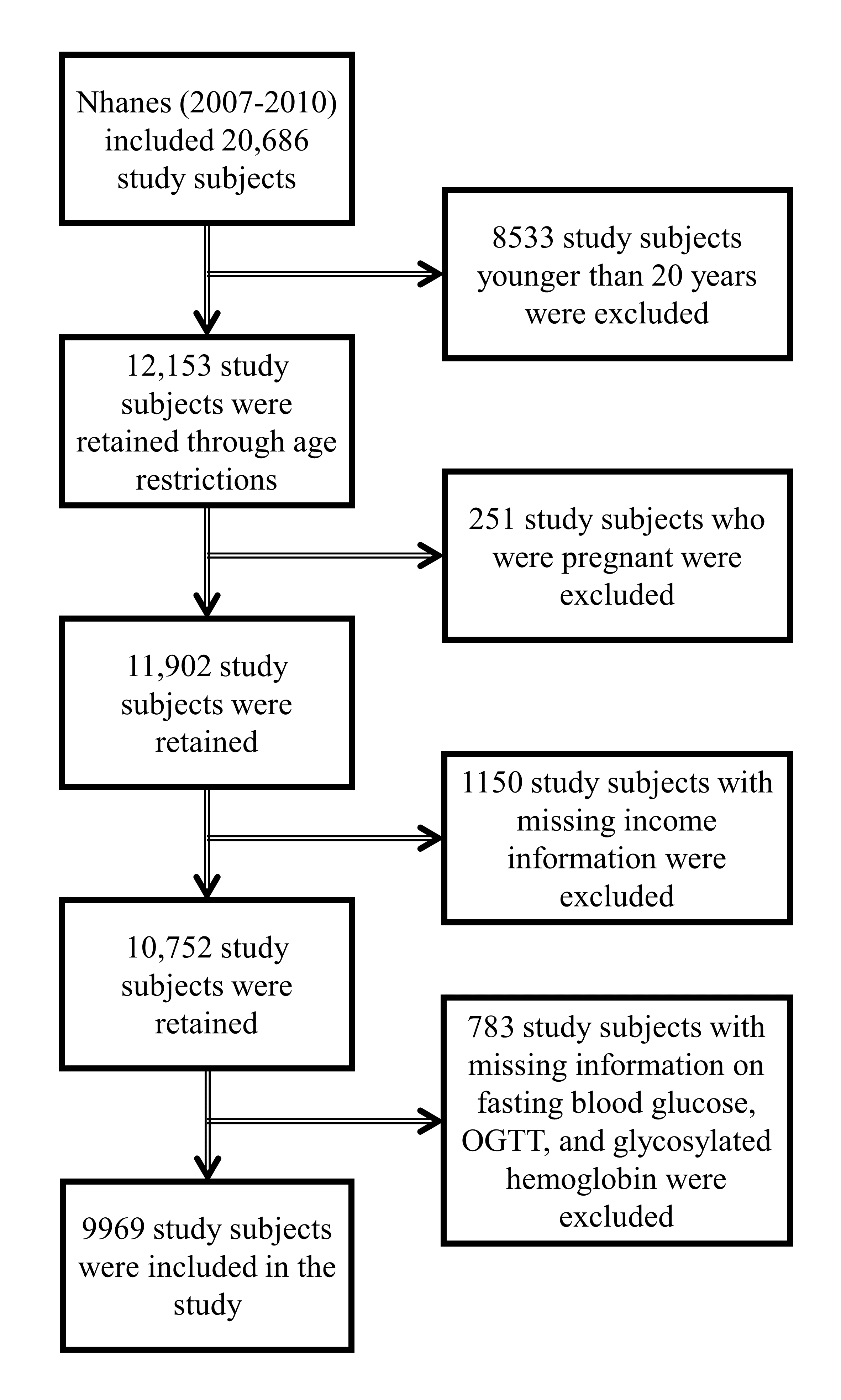
**

**Figure S1.** Schematic diagram of the criteria for inclusion and exclusion of study subjects.

**

**

**Figure S2.** Path hypothesis of mediating effect between socioeconomic status and diabetes.


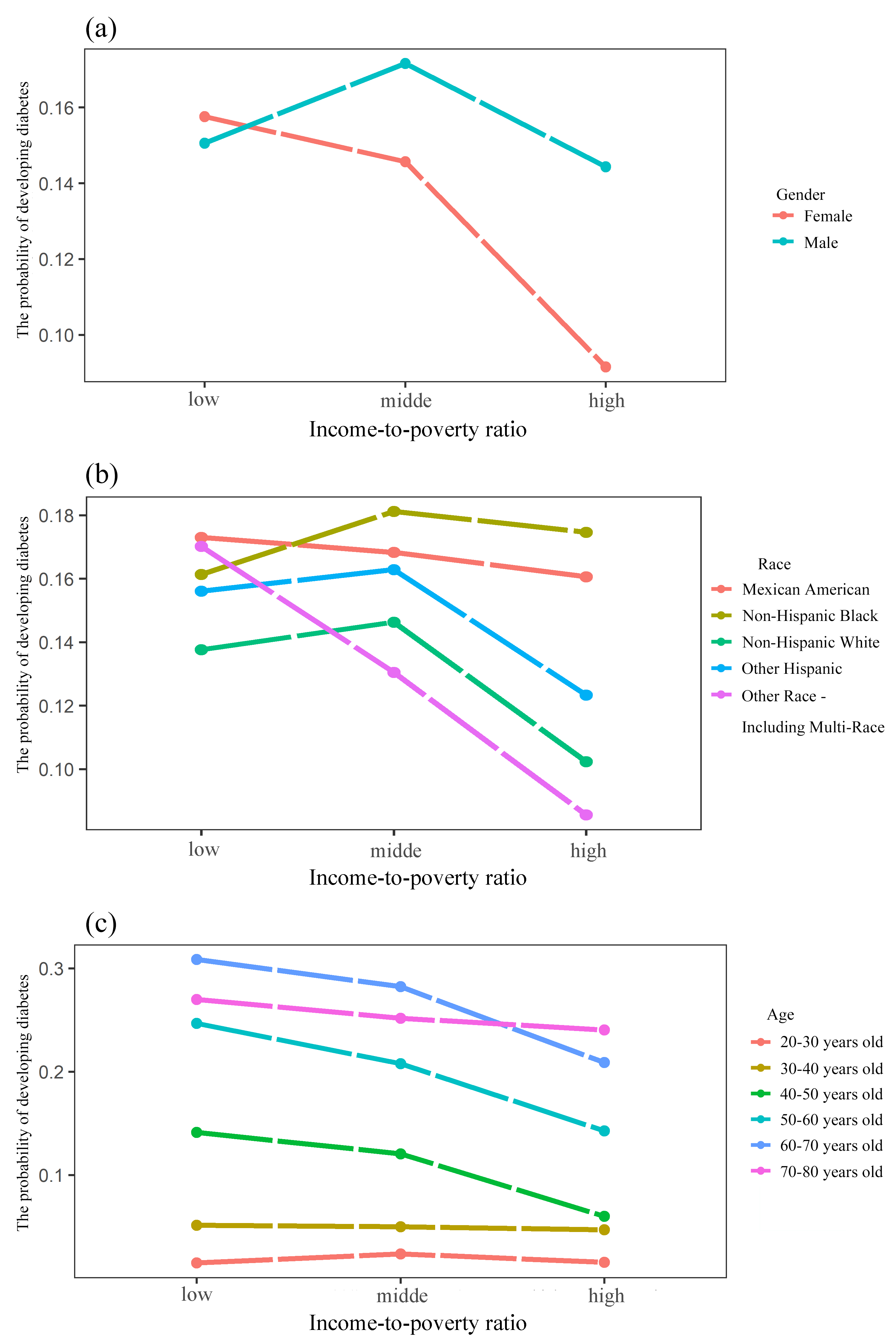


**Figure S3.** Interaction diagram of gender (a), race (b) and age (c) with poverty-to-income ratio respectively.


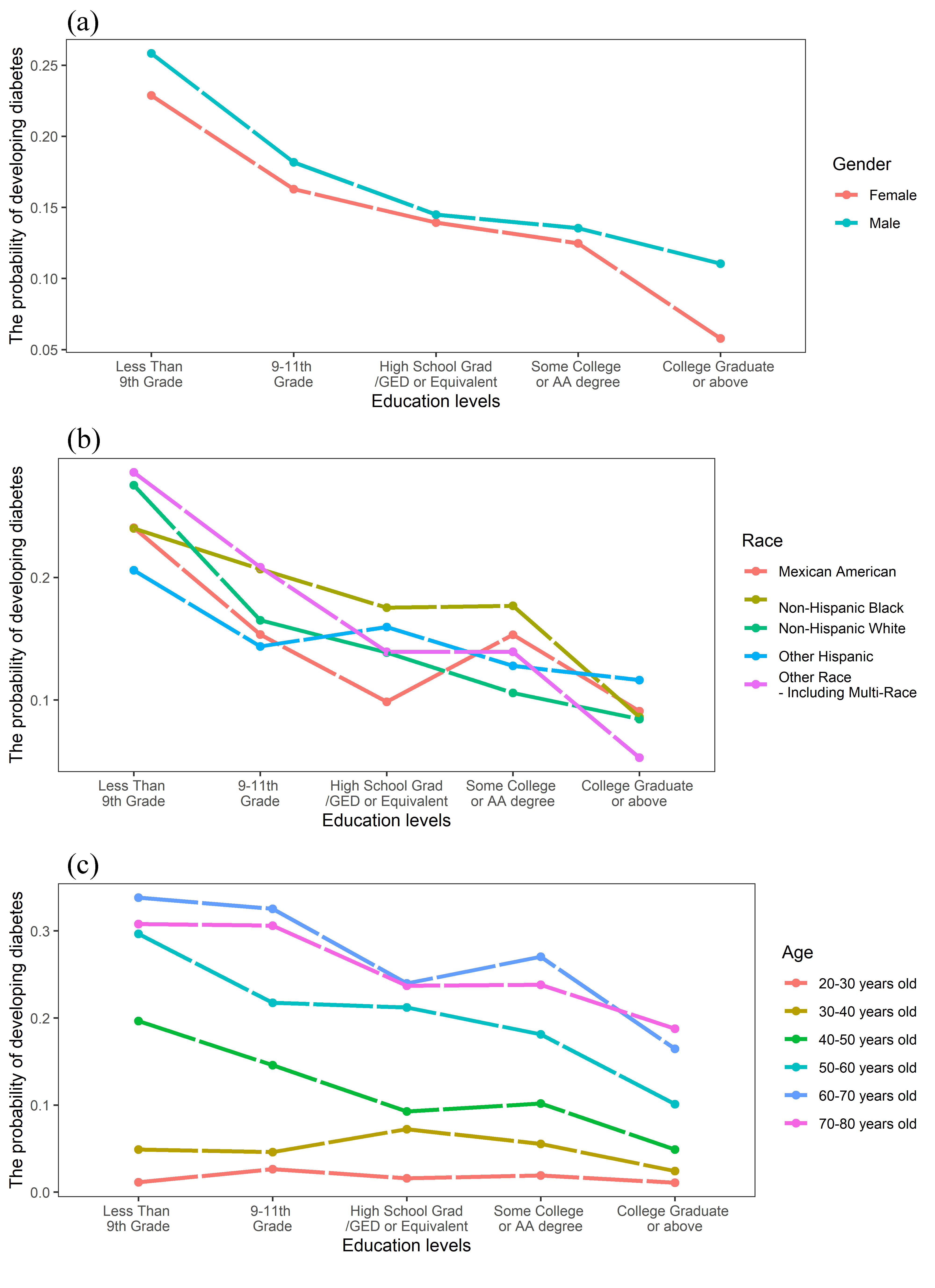


**Figure S4.** Interaction diagram of gender (a), race (b), and age (c) with education levels respectively.


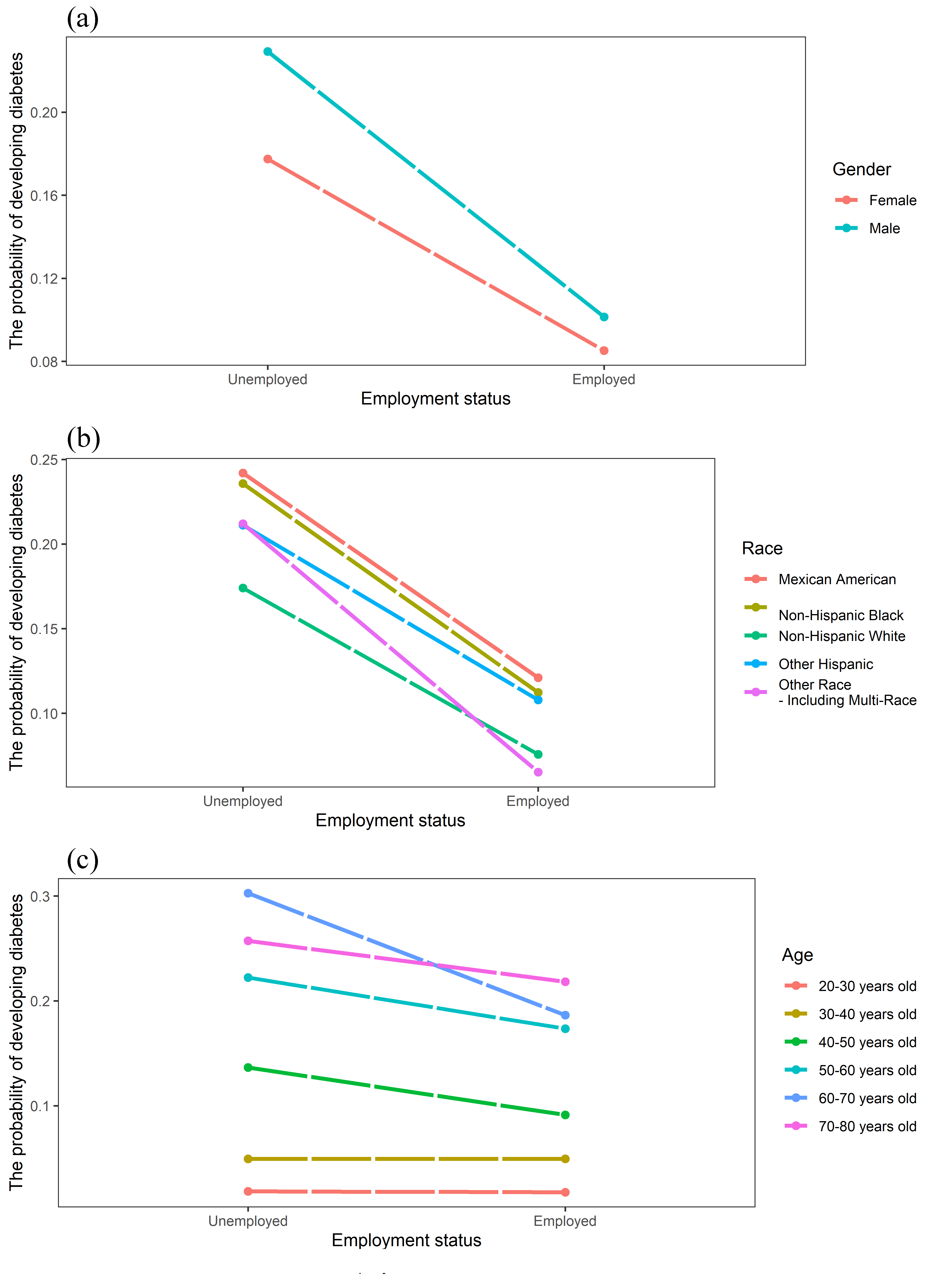


**Figure S5.** Interaction diagram of gender (a), race (b), and age (c) with employment status respectively.

| Supplementary Tables Table S1. Baseline characteristics of the study population by income-to-poverty ratio. | | | | | | | | | |
| --- | --- | --- | --- | --- | --- | --- | --- | --- | --- |
| Characteristics | Total (n= 9969) | | Income-to-poverty ratio ^a^ | | | | | | P-value |
|  |  |  | Low (n=3,354, 33.64%) | | Middle (n=3,378, 33.89%) | | High (n=3,237, 32.47%) | |  |
|  | % | Median (IQR) | % | Median (IQR) | % | Median (IQR) | % | Median (IQR) |  |
| Age |  | 50 (36, 64) |  | 46 (32, 62) |  | 51 (36, 68) |  | 51 (39, 63) | P<0.001 |
| Gender |  |  |  |  |  |  |  |  |  |
| Male | 49.43 |  | 45.14 |  | 50.21 |  | 53.07 |  | P<0.001 |
| Female | 50.57 |  | 54.86 |  | 49.79 |  | 46.93 |  | P<0.001 |
| Race/Ethnicity |  |  |  |  |  |  |  |  | P<0.001 |
| Mexican American | 17.12 |  | 24.12 |  | 18.47 |  | 8.46 |  |  |
| Non-Hispanic Black | 18.23 |  | 17.74 |  | 20.75 |  | 16.10 |  |  |
| Non-Hispanic White | 49.95 |  | 40.31 |  | 46.33 |  | 63.73 |  |  |
| Other Hispanic | 9.90 |  | 12.22 |  | 10.36 |  | 7.01 |  |  |
| Other Race - Including Multi-Race | 4.79 |  | 5.61 |  | 4.09 |  | 4.70 |  |  |
| Less than high school education | 28.82 |  | 47.11 |  | 29.40 |  | 9.27 |  | P<0.001 |
| Smoking |  |  |  |  |  |  |  |  | P<0.001 |
| Current smoker^b^ | 22.23 |  | 32.76 |  | 19.39 |  | 14.27 |  |  |
| Past smoker^c^ | 25.38 |  | 20.47 |  | 27.25 |  | 28.48 |  |  |
| Non-smoker^d^ | 52.39 |  | 46.77 |  | 53.36 |  | 57.25 |  |  |
| High alcohol intake^e^ | 15.59 |  | 19.59 |  | 15.5 |  | 11.46 |  | P<0.001 |
| Low physical activity^f^ | 67.49 |  | 76.51 |  | 69.98 |  | 55.55 |  | P<0.001 |
| A diet lacking fruit^g^ | 3.48 |  | 4.86 |  | 3.64 |  | 1.88 |  | P<0.001 |
| A diet lacking green vegetables^h^ | 6.70 |  | 8.23 |  | 7.08 |  | 4.73 |  | P<0.001 |
| Sedentary time ≥ 3 hours/day | 64.57 |  | 56.59 |  | 62.20 |  | 75.32 |  | P<0.001 |
| Covered by health insurance | 76.05 |  | 60.08 |  | 75.96 |  | 92.68 |  | P<0.001 |
| No healthcare in the past year | 16.29 |  | 20.99 |  | 16.93 |  | 10.75 |  | P<0.001 |
| Fasting blood glucose, mg/dL |  | 101 (93, 112) |  | 101 (93, 113) |  | 101 (93, 113) |  | 100 (93, 109) | P<0.05 |
| Two-hour blood glucose, mg/dL |  | 110 (89, 142) |  | 110 (87, 146) |  | 115 (93, 148) |  | 106 (86, 132) | P<0.001 |
| Glycosylated hemoglobin, % |  | 5.5 (5.2, 5.9) |  | 5.5 (5.2, 5.9) |  | 5.6 (5.3, 6) |  | 5.5 (5.2, 5.8) | P<0.001 |
| Diabetes | 14.45 |  | 15.44 |  | 15.87 |  | 11.96 |  | P<0.01 |

Abbreviations: IQR, interquartile range.

^a^Income-to-poverty ratio is determined by the ratio of household income to the U.S. poverty line and is divided into three levels (≤1.36 (low), 1.37-3.29 (middle), 3.30-5.00 (high)).

^b^Smoked more than 100 cigarettes in a lifetime and still reported daily or occasional smoking at the time of interview.

^c^Smoked more than 100 cigarettes in her lifetime, but still reported quitting smoking in an interview.

^d^Do not smoke more than 100 cigarettes in a lifetime.

^e^Drinking more than 3 alcoholic beverages per month in the 12 months preceding the interview.

^f^Engage in moderate or vigorous recreational physical activity less than 3 times per week.

^g^Reported as having no or very little fruit in the house.

^h^Reported as having no or very few green vegetables in the house.

| Table S2. Baseline characteristics of the study population by education level. | | | | | | | | | | | | | |
| --- | --- | --- | --- | --- | --- | --- | --- | --- | --- | --- | --- | --- | --- |
| Characteristics | Total (n= 9969) | | Education Levels^a^ | | | | | | | | | | P-value |
|  |  |  | Less Than 9th Grade (n=1,216, 12.20%) | | 9-11th Grade (n=1,669, 16.74%) | | High School Grad/GED or Equivalent(n=2,349, 23.56%) | | Some College or AA degree (n=2,708, 27.16%) | | College Graduate or above (n=2,027,20.33%) | |  |
|  | % | Median (IQR) | % | Median (IQR) | % | Median (IQR) | % | Median (IQR) | % | Median (IQR) | % | Median (IQR) |  |
| Age |  | 50 (36, 64) |  | 60 (45, 71) |  | 49 (35, 65) |  | 50 (34, 65) |  | 46 (32, 62) |  | 49 (36, 62) | P<0.001 |
| Gender |  |  |  |  |  |  |  |  |  |  |  |  |  |
| Male | 49.43 |  | 52.08 |  | 48.47 |  | 51.72 |  | 45.53 |  | 51.36 |  | P<0.001 |
| Female | 50.57 |  | 47.92 |  | 51.53 |  | 48.28 |  | 54.47 |  | 48.64 |  | P<0.001 |
| Race/Ethnicity |  |  |  |  |  |  |  |  |  |  |  |  | P<0.001 |
| Mexican American | 17.12 |  | 48.52 |  | 20.31 |  | 13.41 |  | 11.82 |  | 7.05 |  |  |
| Non-Hispanic Black | 18.23 |  | 8.22 |  | 24.93 |  | 19.67 |  | 20.68 |  | 13.76 |  |  |
| Non-Hispanic White | 49.95 |  | 21.55 |  | 41.04 |  | 55.56 |  | 53.47 |  | 63.15 |  |  |
| Other Hispanic | 9.90 |  | 16.86 |  | 10.84 |  | 8.00 |  | 9.53 |  | 7.65 |  |  |
| Other Race - Including Multi-Race | 4.79 |  | 4.85 |  | 2.88 |  | 3.36 |  | 4.51 |  | 8.39 |  |  |
| Smoking |  |  |  |  |  |  |  |  |  |  |  |  | P<0.001 |
| Current smoker^b^ | 22.23 |  | 20.08 |  | 34.59 |  | 27.98 |  | 20.68 |  | 8.73 |  |  |
| Past smoker^c^ | 25.38 |  | 26.26 |  | 24.38 |  | 23.85 |  | 26.18 |  | 26.30 |  |  |
| Non-smoker^d^ | 52.39 |  | 53.66 |  | 41.02 |  | 48.17 |  | 53.14 |  | 64.97 |  |  |
| High alcohol intake^e^ | 15.59 |  | 38.89 |  | 37.85 |  | 31.70 |  | 22.11 |  | 10.62 |  | P<0.001 |
| Low physical activity^f^ | 67.49 |  | 83.55 |  | 80.77 |  | 72.58 |  | 62.81 |  | 47.26 |  | P<0.001 |
| A diet lacking fruit^g^ | 3.48 |  | 4.03 |  | 4.43 |  | 3.32 |  | 3.66 |  | 2.32 |  | P<0.001 |
| A diet lacking green vegetables^h^ | 6.70 |  | 8.66 |  | 6.90 |  | 8.08 |  | 6.38 |  | 4.31 |  | P<0.001 |
| Sedentary time ≥ 3 hours/day | 64.57 |  | 44.24 |  | 57.70 |  | 63.18 |  | 69.17 |  | 77.90 |  | P<0.001 |
| Covered by health insurance | 76.05 |  | 60.53 |  | 66.99 |  | 74.12 |  | 79.39 |  | 90.58 |  | P<0.001 |
| No healthcare in the past year | 16.29 |  | 20.97 |  | 20.19 |  | 17.03 |  | 14.88 |  | 11.30 |  | P<0.001 |
| Fasting blood glucose, mg/dL |  | 101 (93, 112) |  | 105 (97, 120) |  | 103 (95, 115) |  | 102 (94, 113) |  | 99 (92, 108) |  | 99 (92, 107) | P<0.05 |
| Two-hour blood glucose, mg/dL |  | 110 (89, 142) |  | 126.5 (100, 173) |  | 113 (92, 145) |  | 112 (90, 147) |  | 107 (96, 137) |  | 105 (85, 131) | P<0.001 |
| Glycosylated hemoglobin, % |  | 5.5 (5.2, 5.9) |  | 5.7 (5.4, 6.2) |  | 5.6 (5.3, 6) |  | 5.5 (5.3, 5.9) |  | 5.5 (5.2, 5.8) |  | 5.4 (5.2, 5/7) | P<0.001 |
| Diabetes | 14.45 |  | 24.42 |  | 17.20 |  | 14.22 |  | 12.96 |  | 8.49 |  | P<0.01 |

Abbreviations: IQR, interquartile range.

^a^The educational level is divided into five levels: "Less Than 9th Grade", "9-11th Grade", "High School Grad/GED or Equivalent", "Some College or AA degree", and "College Graduate or above".

^b^Smoked more than 100 cigarettes in a lifetime and still reported daily or occasional smoking at the time of interview.

^c^Smoked more than 100 cigarettes in her lifetime, but still reported quitting smoking in an interview.

^f^Engage in moderate or vigorous recreational physical activity less than 3 times per week.

^g^Reported as having no or very little fruit in the house.

^h^Reported as having no or very few green vegetables in the house.

| **Table S3.** Baseline characteristics of the study population by employment status. | | | | | | | |
| --- | --- | --- | --- | --- | --- | --- | --- |
|  | Total (n= 9969) | | Employment Status^a^ | | | | P-value |
| Characteristics |  |  | unemployment (n= 4,737, 47.52%) | | employment (n= 5,232, 52.48%) | |  |
|  | % | Median (IQR) | % | Median (IQR) | % | Median (IQR) |  |
| Age |  | 50 (36, 64) |  | 62 (43, 74) |  | 43 (32, 53) | P<0.001 |
| Gender |  |  |  |  |  |  |  |
| Male | 49.43 |  | 44.10 |  | 54.26 |  | P<0.001 |
| Female | 50.57 |  | 55.90 |  | 45.74 |  | P<0.001 |
| Race/Ethnicity |  |  |  |  |  |  | P<0.001 |
| Mexican American | 17.12 |  | 14.40 |  | 19.59 |  |  |
| Non-Hispanic Black | 18.23 |  | 18.81 |  | 17.70 |  |  |
| Non-Hispanic White | 49.95 |  | 53.51 |  | 46.73 |  |  |
| Other Hispanic | 9.90 |  | 8.70 |  | 10.99 |  |  |
| Other Race - Including Multi-Race | 4.79 |  | 4.58 |  | 4.99 |  |  |
| Smoking |  |  |  |  |  |  | P<0.001 |
| Current smoker^b^ | 22.23 |  | 22.35 |  | 22.11 |  |  |
| Past smoker^c^ | 25.38 |  | 30.29 |  | 20.90 |  |  |
| Non-smoker^d^ | 52.39 |  | 47.36 |  | 56.99 |  |  |
| High alcohol intake^e^ | 15.59 |  | 11.29 |  | 19.48 |  | P<0.001 |
| Low physical activity^f^ | 67.49 |  | 70.64 |  | 64.64 |  | P<0.001 |
| A diet lacking fruit^g^ | 3.48 |  | 3.08 |  | 3.84 |  | P<0.001 |
| A diet lacking green vegetables^h^ | 6.70 |  | 6.71 |  | 6.69 |  | P<0.001 |
| Sedentary time ≥ 3 hours/day | 64.57 |  | 67.70 |  | 61.74 |  | P<0.001 |
| Covered by health insurance | 76.05 |  | 79.92 |  | 72.53 |  | P<0.001 |
| No healthcare in the past year | 16.29 |  | 9.71 |  | 22.25 |  | P<0.001 |
| Fasting blood glucose, mg/dL |  | 101 (93, 112) |  | 103 (95, 116) |  | 99 (92, 107) | P<0.05 |
| Two-hour blood glucose, mg/dL |  | 110 (89, 142) |  | 121 (96, 160) |  | 104.5 (85, 129) | P<0.001 |
| Glycosylated hemoglobin, % |  | 5.5 (5.2, 5.9) |  | 5.6 (5.3, 6.1) |  | 5.4 (5.2, 5.7) | P<0.001 |
| Diabetes | 14.45 |  | 20.03 |  | 9.40 |  | P<0.01 |

Abbreviations: IQR, interquartile range.

^a^The employment status is divided into two levels: "Unemployment", and "employment".

^b^Smoked more than 100 cigarettes in a lifetime and still reported daily or occasional smoking at the time of the interview.

^c^Smoked more than 100 cigarettes in her lifetime, but still reported quitting smoking in an interview.

^d^Do not smoke more than 100 cigarettes in a lifetime.

^e^Drinking more than 3 alcoholic beverages per month in the 12 months preceding the interview.

^f^Engage in moderate or vigorous recreational physical activity less than 3 times per week.

^g^Reported as having no or very little fruit in the house.

^h^Reported as having no or very few green vegetables in the house.
